# A robust bacterial high-throughput screening system to evaluate single nucleotide polymorphisms of human homogentisate 1,2-dioxygenase in the context of alkaptonuria

**DOI:** 10.1038/s41598-022-23702-y

**Published:** 2022-11-14

**Authors:** Sien Lequeue, Jessie Neuckermans, Ine Nulmans, Ulrich Schwaneberg, Tamara Vanhaecke, Joery De Kock

**Affiliations:** 1 Liver Therapy & Evolution Team, In Vitro Toxicology and Dermato-Cosmetology (IVTD) Research Group, Faculty of Medicine and Pharmacy, Vrije Universiteit Brussel, Laarbeeklaan 103, B-1090 Brussels, Belgium; 2 Lehrstuhl Für Biotechnologie, RWTH Aachen University, Worringerweg 3, 52074 Aachen, Germany; 3 In Vitro Liver Disease Modelling Team, In Vitro Toxicology and Dermato-Cosmetology (IVTD) Research Group, Faculty of Medicine and Pharmacy, Vrije Universiteit Brussel, Laarbeeklaan 103, B-1090 Brussels, Belgium

**Keywords:** Metabolic disorders, Bacteria, Drug development, High-throughput screening, Mutation, Biotechnology, Diseases

## Abstract

Alkaptonuria (AKU) is a rare inborn error of metabolism caused by a defective homogentisate 1,2-dioxygenase (HGD), an enzyme involved in the tyrosine degradation pathway. Loss of HGD function leads to the accumulation of homogentisic acid (HGA) in connective body tissues in a process called ochronosis, which results on the long term in an early-onset and severe osteoarthropathy. HGD’s quaternary structure is known to be easily disrupted by missense mutations, which makes them an interesting target for novel treatment strategies that aim to rescue enzyme activity. However, only prediction models are available providing information on a structural basis. Therefore, an *E. coli* based whole-cell screening was developed to evaluate HGD missense variants in 96-well microtiter plates. The screening principle is based on HGD’s ability to convert the oxidation sensitive HGA into maleylacetoacetate. More precisely, catalytic activity could be deduced from pyomelanin absorbance measurements, derived from the auto-oxidation of remaining HGA. Optimized screening conditions comprised several *E. coli* expression strains, varied expression temperatures and varied substrate concentrations. In addition, plate uniformity, signal variability and spatial uniformity were investigated and optimized. Finally, eight HGD missense variants were generated via site-directed mutagenesis and evaluated with the developed high-throughput screening (HTS) assay. For the HTS assay, quality parameters passed the minimum acceptance criterion for Z’ values > 0.4 and single window values > 2. We found that activity percentages *versus* wildtype HGD were 70.37 ± 3.08% (for M368V), 68.78 ± 6.40% (for E42A), 58.15 ± 1.16% (for A122V), 69.07 ± 2.26% (for Y62C), 35.26 ± 1.90% (for G161R), 35.86 ± 1.14% (for P230S), 23.43 ± 4.63% (for G115R) and 19.57 ± 11.00% (for G361R). To conclude, a robust, simple, and cost-effective HTS system was developed to reliably evaluate and distinguish human HGD missense variants by their HGA consumption ability. This HGA quantification assay may lay the foundation for the development of novel treatment strategies for missense variants in AKU.

## Introduction

Inborn errors of metabolism are rare diseases characterized by a defective enzyme in a metabolic pathway. Enzymatic deficiency characteristically results in an accumulation of substrates or their metabolites with often toxic effects^1^. In addition, a metabolic block can eventually occur whereafter reaction products can become deficient. These diseases are in first instance monitored by reducing the concentration of (toxic) substrates in tissues and plasma through enhancing their excretion. On the other hand, deficient reaction products need to be supplemented if these are required for normal growth and development^2^. In this study, we focus on the chronically debilitating disease alkaptonuria (AKU; OMIM #203500). AKU is one of the six tyrosine-related inherited metabolic disorders that are caused by a single dysfunctional hepatic enzyme of the tyrosine degradation pathway^1,3–6^. AKU results from a deficient homogentisate 1,2-dioxygenase (HGD), mainly expressed in liver and kidney cells^1,5,7^. HGD is responsible for the conversion of homogentisic acid (HGA) into maleylacetoacetate (MAA), the third step in the tyrosine degradation pathway. As a result, loss of HGD function blocks tyrosine catabolism leading to the accumulation of HGA in body fluids and urine^8,9^. The latter will cause circulating HGA to oxidize into melanin-like pigment via benzoquinone acetic acid (BQA)^7–10^. This pigment binds preferentially to connective body tissues (*e.g.* cartilage, bone, sclera, etc.) during a process called ochronosis^3,10,11^. Ochronosis and therefore AKU is characterized by premature arthritis, lithiasis, cardiac valve disease, fractures, muscle and tendon ruptures and osteopenia^8,12–14^. In addition to ochronosis, other systemic features can occur including stones (renal, prostatic, salivary, gall bladder), renal damage/failure, respiratory compromise and hearing loss^3^. AKU affects 1 in every 250,000 people worldwide^3,8,10,15,16^. Although, a higher birth prevalence of 1 in every 19,000 people was yet observed in Slovakia and the Dominican Republic. However, recently, an increased prevalence was discovered in Jordan and India^17–23^. AKU is a progressive debilitating disorder for which unfortunately until now only palliative therapies and arthroplasty exist^3,6,10^. Since 2020, Nitisinone ([2-(2-nitro-4-trifluoromethylbenzoyl)cyclohexane-1,3-dione; (NTBC)], a triketone herbicide that has been approved by the Food and Drug Administration for over 20 years to treat type-1 hereditary tyrosinemia (HT1-#276700)^6,8^, was also granted marketing authorization for AKU as it is known to decrease serum and urine HGA levels by 95%^10,24,25^. NTBC is a competitive, reversible and potent inhibitor of 4-hydroxyphenyl-pyruvate dioxygenase, resulting in a block upstream of HGD in the tyrosine degradation pathway preventing the accumulation of HGA^3,10,13^. Although, NTBC might be of great advantage for AKU patients, it is unfortunately also associated with adverse effects. Indeed, increased tyrosine serum levels (hypertyrosinemia), corneal keratopathy as well as ocular irritation and photophobia have been associated with NTBC treatment^26–28^. Even more worryingly, a relationship was seen between elevated tyrosine plasma levels and impaired cognitive function upon long-term NTBC treatment of HT-1 patients^29^. Moreover, recently, increased tyrosine concentrations were found in murine and human tissues (*i.e.* acquired tyrosinosis) after NTBC treatment, however its impact on organ function needs further investigation^30^. Additionally, metabolites of dopaminergic and adrenergic neurotransmitters from which tyrosine is the precursor, have previously been shown to play a significant role in the development of intellectual impairment, but still its cause has to be elucidated^31^. Dietary restriction of tyrosine and phenylalanine was found to manage NTBC-induced hypertyrosinemia in AKU patients as well as in mice^28^. Although NTBC offers multiple benefits in the context of AKU, it does not restore the enzymatic defect inflicted by the disease nor does it cure the disease. The aforementioned facts clearly emphasize the need for improving the current treatment strategy for AKU and focus on alternative and complementary treatment options^14^.

AKU may be a rare disease, albeit it appeared to be the first case of recessive inheritance discovered in humans by Garrod about more than a century ago^32^. In 1958, it has been demonstrated by La Du et al. that AKU patients fail to synthesize an active form of the hepatic HGD enzyme^33^. Moreover, Fernández-Cañón et al. discovered that loss of HGD function is induced by homozygous or compound heterozygous mutations within the HGD gene^34^. The HGD gene consists of 54 363 base pairs (located on chromosome 3q13.33), composes of 14 exons and codes for the HGD protein protomer of 445 amino acids^11,17,25^. HGD has as a hexamer, composed of two disk-like trimers, a highly complex quaternary structure and sophisticated catalytic mechanism^11,17,25,35^. Furthermore, it has been shown that this delicate structure can be easily disrupted by missense mutations ^11,25,35^. At present, 251 unique AKU-causing variants have been reported and summarized in the HGD mutation database^36^. Moreover, missense variants denote approximately for 65% of all known AKU-causing variants^11,17^ and can affect the HGD enzyme activity by three molecular mechanisms: (i) stability reduction of individual protomers, (ii) disruption of protomer-protomer interactions or (iii) genetic modifications in the catalytic site of the enzyme^11,25^. So far, the impact of these missense variants on protein stability, folding and conformational flexibility can be analyzed by biophysical tools (*i.e.* mCSM-Stability, DUET and DynaMut)^37^. However, no methods exist to estimate the possible residual activities of HGD missense variants^14^. Here, a robust, fast and inexpensive whole-cell bioassay was developed that enables a rapid screening of HGD missense variants to estimate their residual enzyme activities.

With the purpose to design a reliable high-throughput screening (HTS) assay based upon HGD activity, *Escherichia coli* (*E. coli)* is the preferred choice as host organism for (re-)screening of recombinant proteins as these cells allow easy manipulation, grow rapidly and can be cultured inexpensively^38,39^. Moreover, *E. coli* cloning systems have been developed over time in such a way that they can be used with high efficiency, fidelity, and reliability, making them widely used in both commercial and academic settings for the large scale screening of recombinant proteins^38^. The developed HTS assay is based on the evaluation of human HGD variants by their HGA consumption ability in a whole cell screening setup after their exogenous HGD expression. More precisely, HGD converts the oxidation sensitive HGA into MAA until complete enzyme saturation occurs. Subsequently, excess HGA will accumulate and auto-oxidize into a brown pyomelanin-like pigment which can spectrophotometrically be quantified. Our newly developed colorimetric HTS assay is able to quantify the ability to consume HGA and, as such, HGD residual activities can be deduced from pyomelanin absorbance measurements. Furthermore, the indirect measurement of pyomelanin is of high clinical relevance for AKU since its progression is characterized by the formation of ochronotic pigment leading to its disease outcome whereas the direct measurement of MAA would only provide information on the intrinsic metabolic HGD activity. In addition, eight missense variants (G161R, A122V, E42A, M368V, Y62C, P230S, G115R and G361R) were generated via site-directed mutagenesis (SDM) to demonstrate the method’s ability to evaluate the residual activity of real-life variants, compared to wildtype (WT) HGD. G161R, a variant mainly observed in Slovakia, results in protomer destabilization and its residual enzyme activity is estimated at less than one percent compared to WT HGD. In addition, variant A122V is mainly observed in Jordan whereas the M368V variant is most frequently observed in Europe. The latter two are responsible for quaternary structure disruption of the hexameric active site and their specific HGD activities were estimated to be approximately 30 percent compared to its WT counterpart^25^. Moreover, missense variants E42A and Y62C have residual enzyme activities estimated at 29 and 22.5 percent, respectively, and cause protomer destabilization as well as hexamer disruption^25,40^. Finally, variants G115R and G361R cause both protomer destabilization but here, no residual enzyme activities can be provided as they have not been reported in literature so far^36^. Altogether, this bacterial whole-cell HTS assay was developed with the objective to analyse the residual HGD activity of AKU-causing missense variants in the context of AKU. The availability of such an assay is of great importance for the development of new treatment strategies for selected variants in AKU, for instance, small molecule libraries exist that might help with structural stability to rescue partial or total enzyme activity^35,41–43^.

## Results

### Determination of ideal expression conditions of human HGD in *E. coli*

**Figure 1:**
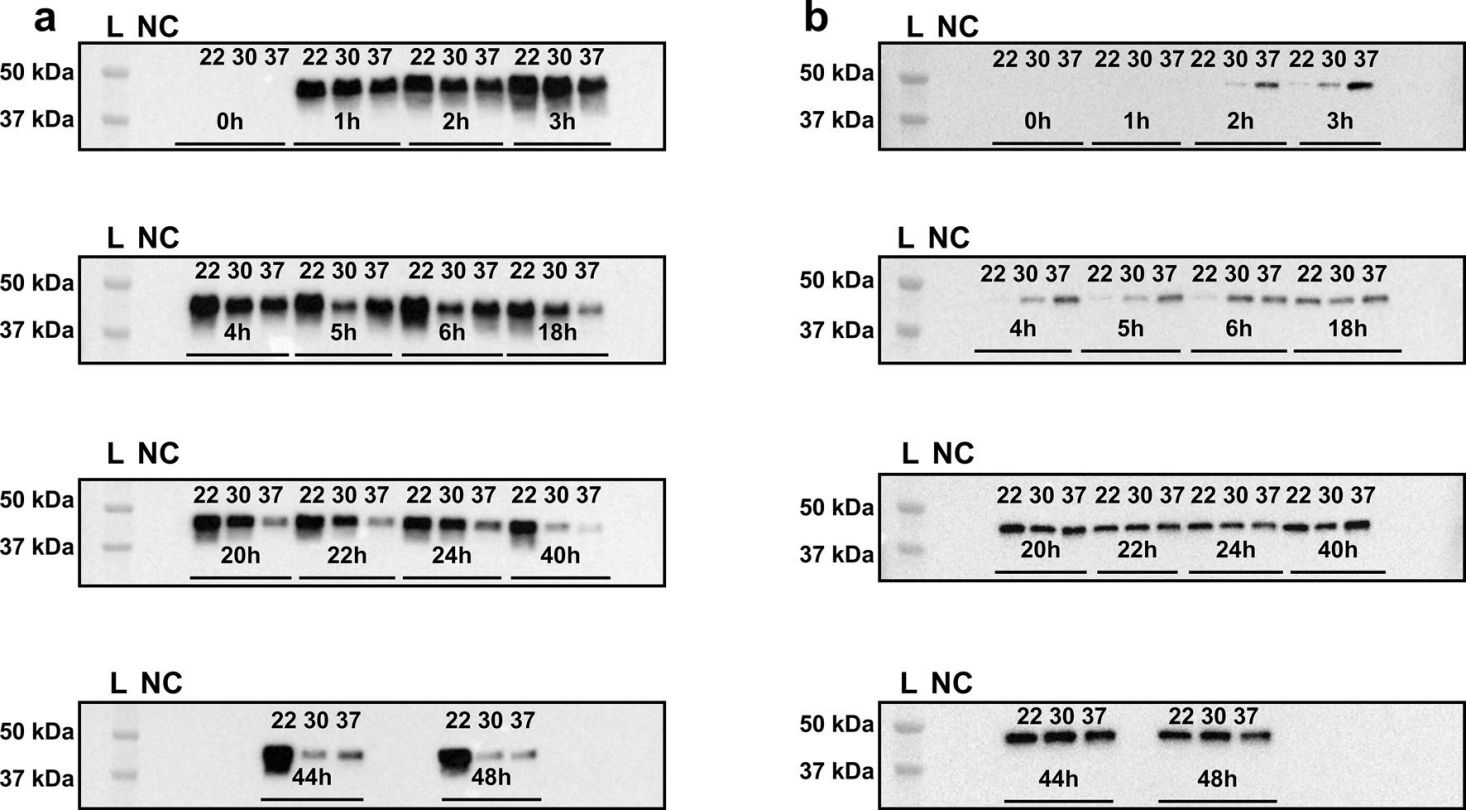
Protein expression of human HGD was evaluated in both *E. coli* BL21 (DE3) (**a**) and *E. coli* C43 (DE3) (**b**) strain and for different expression temperatures (22 °C, 30 °C and 37 °C) by western blot analysis to select the appropriate strain and temperature for reliable expression of human HGD. For both *E. coli* strains, a molecular weight band at 49 kDa was observed. Human HGD expression was higher in *E. coli* BL21 (DE3) compared to *E. coli* C43 (DE3). In *E. coli* BL21 (DE3), an obvious band at the expected molecular weight was observed for the three assessed temperatures from one hour after IPTG addition onwards (**a**). For *E. coli* C43 (DE3), a subtle band at the expected molecular weight was visible two hours post-IPTG addition and became more prominent after 20 h, but protein expression was still found to be lower compared to *E. coli* BL21 (DE3). The latter confirms a lower and more delayed protein expression at the three evaluated temperatures (**b**). Uncropped blots are available as Supplementary Information in Supplementary Fig. S1a (for *E. coli* BL21 (DE3)) and Fig. S1b (for *E. coli* C43 (DE3)).

**Figure 2:**
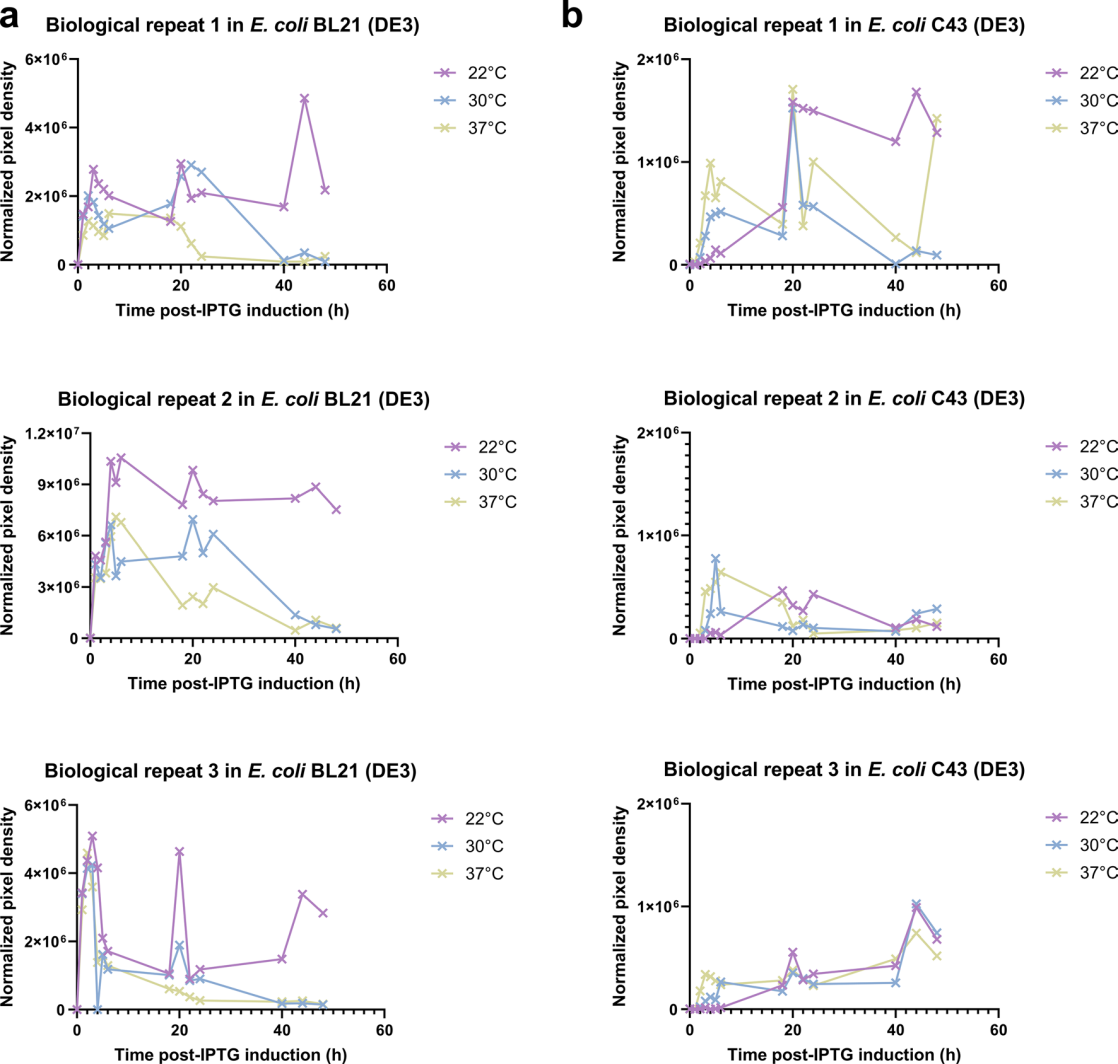
Protein expression conditions of human HGD were evaluated in both *E. coli* BL21 (DE3) (**a**) and *E. coli* C43 (DE3) (**b**) strain. Three different expression temperatures (22 °C, 30 °C and 37 °C) were simultaneously evaluated to select the appropriate temperature for reliable human HGD protein expression. (**a–b**) Samples of the supernatant fraction were evaluated via western blot analysis and normalized pixel densities were compared between biological repeats in triplicate, two *E. coli* strains and three temperatures. The highest and most reliable HGD protein expression was achieved in *E. coli* BL21 (DE3) (**a**), whereas HGD expression was much lower in *E. coli* C43 (DE3) (**b**). Additionally, expression temperatures 22 °C and 30 °C achieved the highest and most stable protein expression in both strains, while HGD was only mediocrely expressed at 37 °C.

In order to develop a robust bacterial HTS system that can distinguish single nucleotide polymorphisms of human HGD enzymes, the optimal expression conditions in *E. coli* as host organism had to be determined. In that objective, protein expression experiments were conducted in both *E. coli* BL21 (DE3) and its equivalent C43 (DE3) strains at different temperatures (22 °C, 30 °C, 37 °C) to determine the best suited strain and temperature for high and reliable human HGD expression. Hence, *E. coli* BL21 (DE3) and *E. coli* C43 (DE3) cells containing both the pET42b-HGD plasmid were pre-grown and protein expression was induced as described in the Materials and Methods section. Subsequently, cultures were incubated at different expression temperatures (22 °C, 30 °C and 37 °C), samples were isolated at regular time intervals post-Isopropyl β-D-1-thiogalactopyranoside (IPTG) addition and supernatant fractions were thereafter evaluated via western blot analyses. For both *E. coli* strains, a molecular weight band at 49 kDa could be observed after western blot analysis (Fig. 1), though, human HGD expression was higher in *E. coli* BL21 (DE3) compared to *E. coli* C43 (DE3) (Fig. 2). Moreover, in *E. coli* BL21 (DE3), an obvious band at the expected molecular weight could be observed for the three assessed temperatures only one hour after induction of protein expression (Fig. 1a), whilst this was not the case for *E. coli* C43 (DE3) whereby only a subtle band at the expected molecular weight was visible two hours post-IPTG addition. Even, after 20 h post-IPTG addition, a more prominent band at the expected molecular weight was observed, but still lower compared to *E. coli* BL21 (DE3). This indicates that HGD protein expression was more delayed at the three evaluated temperatures in *E. coli* C43 (DE3) compared to *E. coli* BL21 (DE3) (Fig. 1b). In *E. coli* BL21 (DE3), one could observe that protein expression was the lowest at 37 °C, whilst the highest at 22 °C and 30 °C (Fig. 2a). However, HGD protein expression decreased at 30 °C after 24 h post-IPTG addition, as opposed to 22 °C where a stable protein expression was still guaranteed 48 h after IPTG induction. Based on these results, expression strain *E. coli* BL21 (DE3) and expression temperatures 22 °C and 30 °C were taken into consideration for further optimization of the assay conditions.

### In vitro pyomelanin formation is complete 24 h post-IPTG induction

**Figure 3:**
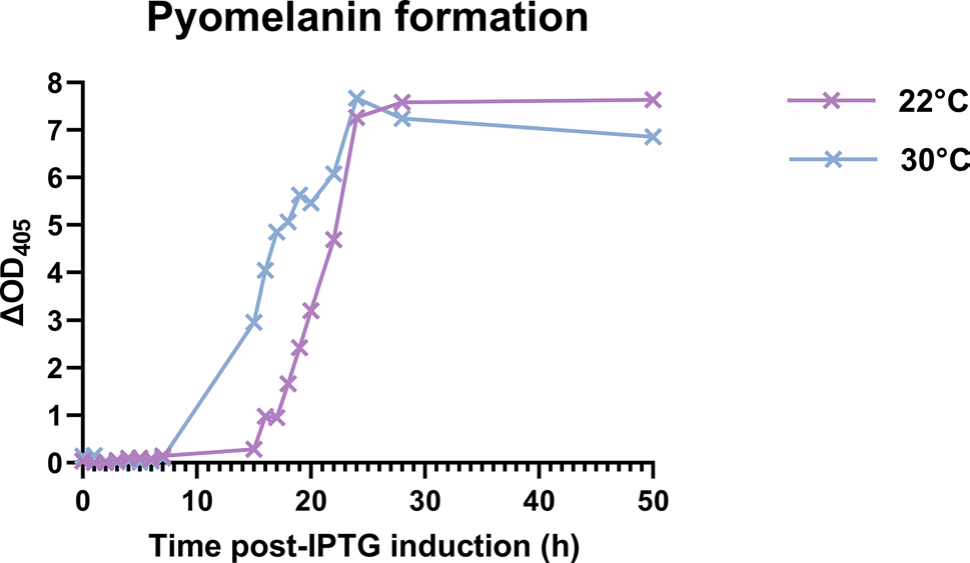
Pyomelanin absorbance measurements (∆OD_405_ ~ amount of HGA consumed by HGD) in function of time post-IPTG induction. After 24 h, the auto-oxidation and self-polymerization of HGA into pyomelanin has reached a steady-state for both expression temperatures. To conclude, OD_405_ endpoint readouts should be performed at the earliest 24 h after induction with IPTG.

The auto-oxidation and self-polymerization process of HGA into pyomelanin should be completed before OD_405_ end-point readouts can be measured. Therefore, a subsequent experiment was conducted to determine at which time point, after the addition of IPTG, pyomelanin formation is complete. In that context, *E. coli* BL21 (DE3) cells containing pET42b-HGD plasmid and the empty vector (EV) control were pre-grown and protein expression was induced as described in the Materials and Methods section. Subsequently, pyomelanin absorbance measurements were performed at regular time intervals to monitor complete auto-oxidation of HGA into melanin-like pigment via BQA. In Fig. 3, ∆OD_405_ (OD_405_ EV – OD_405_ HGD; amount of HGA consumed by HGD) is plotted as a function of time and indicates that 24 h after IPTG induction, HGA’s auto-oxidation and self-polymerization process was stabilized for both expression temperatures. Briefly, this suggests that end-point readouts should be monitored at the earliest 24 h after the addition of IPTG.

### HGA concentration of 2.5 mM provides an ideal signal separation window

**Figure 4:**
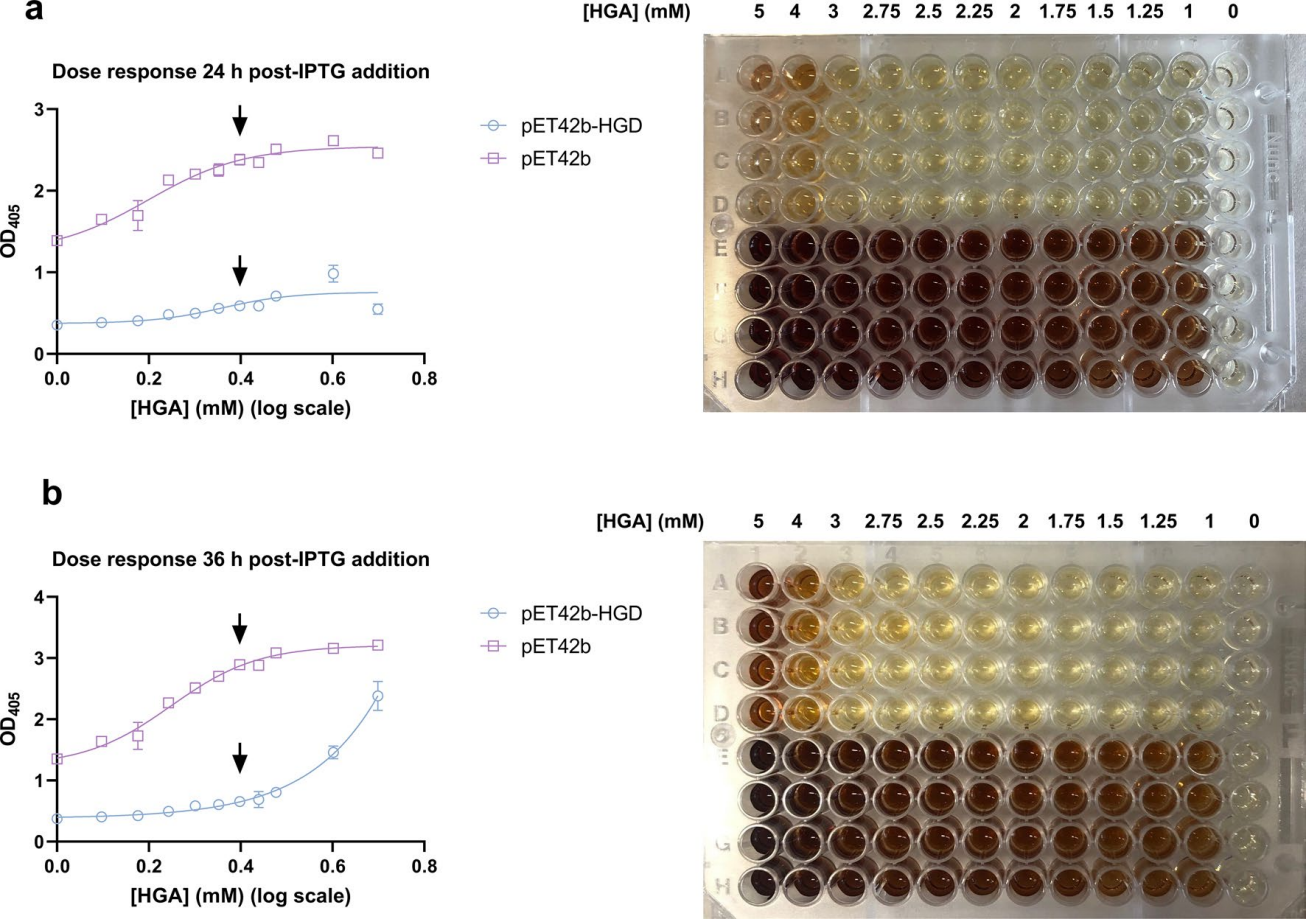
Dose response experiment. *E. coli* BL21 (DE3) cells containing pET42b-HGD plasmid and the EV control were pre-grown and protein expression was induced as described in the Materials and Methods section. Both cultures were supplemented with different HGA concentrations ranging from 0 to 5 mM. After 24 h (**a**) and 36 h (**b**) post-IPTG addition, OD_405_ was measured. An ideal separation window between high and low control signals could be observed when a substrate concentration of 2.5 mM HGA was used.

To obtain a good signal separation window for pyomelanin measurements between a low signal (HGD WT), a high signal (EV) and HGD variants, a dose response experiment with HGA was performed. Therefore, *E. coli* BL21 (DE3) cells containing pET42b-HGD plasmid and the EV control were pre-grown and protein expression was induced as described in the Materials & Methods section. The 96 well-plate was filled according to Fig. 4a and b (right). Firstly, pyomelanin absorbance was measured after 24 h (Fig. 4a left) post-IPTG addition. Secondly, pyomelanin formation was measured after 36 h (Fig. 4b left) to guarantee complete auto-oxidation and self-polymerization of HGA into pyomelanin for the higher HGA concentrations as well. An ideal signal window (SW) between high and low control signals was observed when a substrate concentration of 2.5 mM HGA was used.

### Assay robustness can be guaranteed for expression temperature 30 °C, but not for 22 °C, with minor edge and drift effects

An assay was developed with the intention to rank HGD variants based upon their HGA consumption activity. Therefore, in order to ensure a high-throughput, experiments were performed in 96-well microtiter plate (MTP) format to investigate the plate uniformity and assay robustness.

#### Plate uniformity and signal variability assessment

**Figure 5:**
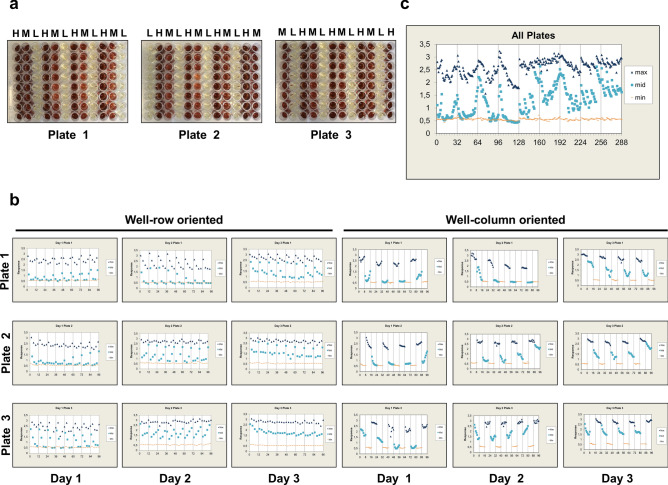
(**a**) To assess the plate uniformity and separation of the signals in our HTS assay at an expression temperature 22 °C; a plate uniformity test was conducted at maximum, medium and minimum signals—0%, 37% and 100% residual HGD activity—in an interleaved signal format to guarantee that the SW is capable of ranking HGD missense variants during screening. All three signals were distributed on each plate but varied systematically so that on all plates, on a given day, each signal is measured on each plate to detect edge or drift effects (3 plates, 3 days). (**b**, **c**) The obtained signals were also plotted on a scatter plot to reveal patterns of drift or edge effect or other systemic sources of variability. Signals were plotted against well number, first in a column-wise fashion then in row-wise fashion and significant (> 20%) edge or drift effect were observed indicating that under the applied conditions, no reliable results can be obtained.

**Figure 6:**
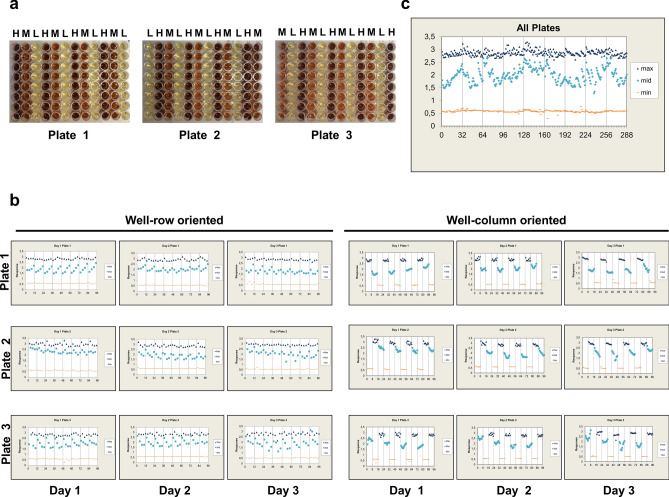
(**a**) To assess the plate uniformity and separation of the signals in our HTS assay at an expression temperature 30 °C; a plate uniformity test was conducted at maximum, medium and minimum signals—0%, 37% and 100% residual HGD activity—in an interleaved signal format to guarantee that the SW is capable of ranking HGD missense variants during screening. All three signals were distributed on each plate but varied systematically so that on all plates, on a given day, each signal is measured on each plate to detect edge or drift effects (3 plates, 3 days). (**b**, **c**) The obtained signals were also plotted on a scatter plot to reveal patterns of drift or edge effect or other systemic sources of variability. Signals were plotted against well number, first in a column-wise fashion then in a row-wise fashion. Maximum row and column drift for Max signals were 6.47% and 9.62%, respectively. For the Mid signal, we found a significant edge effect as higher signals were observed at the outer columns of the 96-well plate with decreasing intensity moving towards the middle columns of the plate. It is thus recommended to avoid columns 1 and 12 for screening of HGD enzyme variants. Respective Max and Min control signals can still be sampled at these locations. Some calculated drift effects for the Mid signal exceeded the > 20% criteria, but effects were only seen on a single plate or at a few plates and no predominant pattern was observed and can be considered insignificant.

The assay validation experiments were run over three days and three individual plates were processed each day to assess the plate uniformity and separation of the signals. Moreover, uniformity tests are always conducted at the maximum and minimum control signals to guarantee that the SW is sufficient to distinguish different HGD missense variants during screening^44^. The variability tests were performed on three different assay readouts mimicking maximum, medium and the minimum signal. Here, the maximum or highest signal corresponded to the signal obtained from the EV culture as in that case all HGA will be available for auto-oxidation into the brownish melanin-like pigment. The minimum or lowest signal corresponded to the signal obtained from the HGD WT culture since here only the remaining HGA will be auto-oxidized into pyomelanin as mostly all HGA will be converted by HGD into MAA. The medium signal corresponded to the signal obtained from the M368V culture. According to literature this variant has a residual human HGD activity of ± 37%^25^ and therefore it was expected that this signal lies between the high and low control signals. Furthermore, all three signals were distributed on each plate in an interleaved fashion but varied systematically so that on all plates, on a given day, each signal is measured on each plate to detect edge or drift effects (Figs. 5a, 6a). This was performed to ensure that all three plates measured on a given day contain signals according to the following order: “high-medium–low” (plate 1), “low–high-medium” (plate 2), “medium–low-high” (plate 3). Firstly, the plate uniformity in 96-well format was evaluated for expression temperature 22 °C and the data is shown in Table 1. These results were used to perform calculations to provide additional information on assay parameters to evaluate the overall quality and separation of the assay control data as well as the plate-to-plate and day-to-day variation. First of all, coefficient of variation (CV) of each signal (*i.e.* maximum, medium and minimum) were calculated and all CV values should pass the < 20% criteria since HTS assays with CV values < 20% are routinely and successfully employed in screening enzyme variant libraries^45,46^. The maximum signal CV’s were between intervals 1.5–6.3%, midpoint signal CV’s were between intervals 3.1–16.6% and minimum signal CV’s were between intervals 1.3–2.3%. Consequently, all CV values pass the < 20% criteria if 10 repeats per equivalent signal were considered. Average plate Z’ values and SW values (Eqs. 2 and 3) were 0.88 ± 0.06 and 31.51 ± 15.67, respectively. Moreover, both parameters pass the minimum acceptance criterion for Z’ values > 0.4 and SW values > 2. However, normalized mid signal (mid %) standard deviations (SD’s) below 20% and within-day fold shifts as well as average between day fold shifts < 2 acceptance criteria were not fulfilled. This indicates that assay robustness cannot be guaranteed under the previously described conditions, possibly due to the fact that the auto-oxidation and self-polymerization process of HGA into pyomelanin was not complete in the 96-well plate at 22 °C. In analogy, assay robustness was also evaluated for expression temperature 30 °C and the data is shown in Table 2. The maximum signal CV’s were between intervals 1.8–3.3%, midpoint signal CV’s were between intervals 5.6–15.8% and minimum signal CV’s were between intervals 1.3–5.2%. Consequently, all CV values pass the < 20% criteria if two repeats per equivalent signal were considered. Average plate Z’ factors and SW values (Eqs. 2 and 3) were 0.88 ± 0.02 and 28.61 ± 6.93, respectively. Both parameters pass the minimum acceptance criterion for Z’ values > 0.4 and SW values > 2. Furthermore, normalized mid signal (mid%) SD’s were below 20% and on scale. Within-day fold shifts and between day fold shifts were < 2 indicating that assay robustness can be guaranteed at an expression temperature of 30 °C.

**Table 1:** Data of the (intra) plate uniformity study for expression temperature 22 °C. H: high signal; M: medium signal; L: low signal; SD: standard deviation; CV: coefficient of variation; Mid%: normalised mid signal; SW: signal window; Z’: Z’ factor. All Max (high) and Mid signal (unnormalized) CV’s < 20%. All min (Low) SD’s < Min (Max (High) SD, mid SD). All SW’s > 2 and Z’ > 0.4. Z’ and CV’s were calculated taken in consideration 10 repeats per equivalent signal. Normalized mid signal (mid %) SD’s below 20 and within-day fold shifts as well as average between-day fold shifts < 2 acceptance criteria were not fulfilled suggesting that under the prescribed conditions, assay robustness could not be guaranteed, possibly due to the fact that pyomelanin formation was not complete when expression temperature 22 °C was used.

Day	Plate	Type	Mean	SD	CV	Mid%	SW	Z′
1	1	H	2.42	0.24	3.2	14.38 ± 15.89	20.78	0.86
		M	0.83	0.29	11.2			
		L	0.56	0.02	1.3			
	2	H	2.40	0.22	2.9	8.79 ± 5.90	23.27	0.88
		M	0.73	0.11	4.7			
		L	0.57	0.02	1.3			
	3	H	2.42	0.32	4.1	29.77 ± 31.12	15.48	0.82
		M	1.11	0.58	16.6			
		L	0.55	0.03	1.7			
2	1	H	2.27	0.46	6.3	0.86 ± 6.73	9.03	0.74
		M	0.70	0.43	19.4			
		L	0.51	0.02	1.4			
	2	H	2.71	0.13	1.5	36.20 ± 26.18	48.04	0.93
		M	1.34	0.56	13.2			
		L	0.57	0.02	1.3			
	3	H	2.86	0.15	1.7	51.57 ± 17.40	44.45	0.92
		M	1.74	0.40	7.3			
		L	0.56	0.04	2.3			
3	1	H	2.69	0.23	2.7	42.71 ± 22.62	26.57	0.89
		M	1.46	0.49	10.6			
		L	0.54	0.03	1.7			
	2	H	2.74	0.14	1.6	47.95 ± 20.53	45.60	0.93
		M	1.61	0.45	8.7			
		L	0.57	0.03	1.4			
	3	H	2.78	0.13	1.5	51.91 ± 7.64	50.40	0.93
		M	1.71	0.17	3.1			
		L	0.57	0.03	1.6

**Table 2:** Data of the (intra) plate uniformity study for expression temperature 30 °C. H: high signal; M: medium signal; L: low signal; SD: standard deviation; CV: coefficient of variation; Mid%: normalised mid signal; SW: signal window; Z’: Z’ factor. All Max (high) and Mid signal (unnormalized) CV’s < 20%. All normalized mid signal (mid%) SD’s < 20. All min (Low) SD’s < Min (Max (High) SD, mid SD). All SW’s > 2 and Z’ > 0.4. Z’ and CV’s were calculated taken in consideration two repeats per HGD variant. Within-day fold shifts and between-day fold shifts were below two indicating that assay robustness can be guaranteed when expression temperature 30 °C is used.

Day	Plate	Type	Mean	SD	CV	Mid%	SW	Z′
1	1	H	2.82	0.08	2.0	57.36 ± 11.8	36.35	0.90
		M	1.86	0.26	10.0			
		L	0.58	0.03	3.4			
	2	H	2.87	0.12	3.1	55.08 ± 10.99	22.50	0.87
		M	1.85	0.25	9.5			
		L	0.61	0.01	1.3			
	3	H	2.82	0.10	2.5	65.00 ± 14.87	27.82	0.89
		M	2.04	0.33	11.5			
		L	0.58	0.01	1.5			
2	1	H	2.91	0.12	2.8	62.83 ± 7.66	25.40	0.89
		M	2.03	0.18	6.3			
		L	0.55	0.01	1.4			
	2	H	2.97	0.14	3.3	73.00 ± 7.72	20.13	0.84
		M	2.33	0.18	5.6			
		L	0.59	0.04	5.2			
	3	H	2.83	0.11	2.8	63.22 ± 13.82	25.11	0.88
		M	2.01	0.31	10.9			
		L	0.60	0.02	2.0			
3	1	H	2.84	0.09	2.1	54.83 ± 9.82	33.72	0.90
		M	1.77	0.21	8.3			
		L	0.57	0.03	3.3			
	2	H	2.92	0.07	1.8	62.94 ± 13.00	41.11	0.91
		M	2.04	0.31	10.7			
		L	0.55	0.03	3.3			
	3	H	2.81	0.11	2.8	62.10 ± 18.97	25.35	0.88
		M	1.94	0.43	15.8			
		L	0.52	0.01	1.9			

#### Spatial uniformity assessment

According to the HTS Assay Validation Guidelines, the overall requirement for HTS systems is that plates do not show material edge or drift effects whereby drift or edge effects < 20% can be classified as insignificant^44^. First of all, an interleaved plate lay-out was used to capture edge effects caused by the applied incubation conditions (*e.g.* temperature gradients) or to detect other systemic errors as drift. Significant trends in Max and Mid signals should be assessed from left-to-right and top-to-bottom to assess drift effects. When expression temperature 22 °C was used, significant column as well as row drift effects were observed for Max and Mid signals possibly due to incomplete formation of pyomelanin at this temperature. Therefore, to significantly reduce plate edge and drift effects, the spatial uniformity assessment was evaluated for expression temperature 30 °C. Here, maximum row and column drift for Max signals were 6.47% and 9.62%, respectively. For the Mid signal, we found a significant edge effect as higher signals were observed at the outer columns of the 96-well plate with decreasing intensity moving towards the columns located at the middle of the plate. This can be due to temperature gradients whereby higher temperatures at the outer wells of the plate are reached. Therefore, it is recommended to avoid columns 1 and 12 for the analysis of HGD missense variants. However, respective Max and Min control signals can still be sampled at these locations. Some of the calculated drift effects for the Mid signal exceeded the > 20% criteria, but effects were only seen on a single plate or at a few plates and no predominant pattern was observed. Therefore, these effects can be considered insignificant. Additionally, the obtained signals were also plotted on a scatter plot to reveal patterns of drift or edge effect or other systemic sources of variability. Therefore, signals are plotted against well number, first in a column-wise fashion then in a row-wise fashion for both expression temperatures (Figs. 5b, c and 6b, c).

##### The bacterial whole cell assay is capable of defining the residual HGA consumption capacity of human HGD missense variants

**Figure 7:**
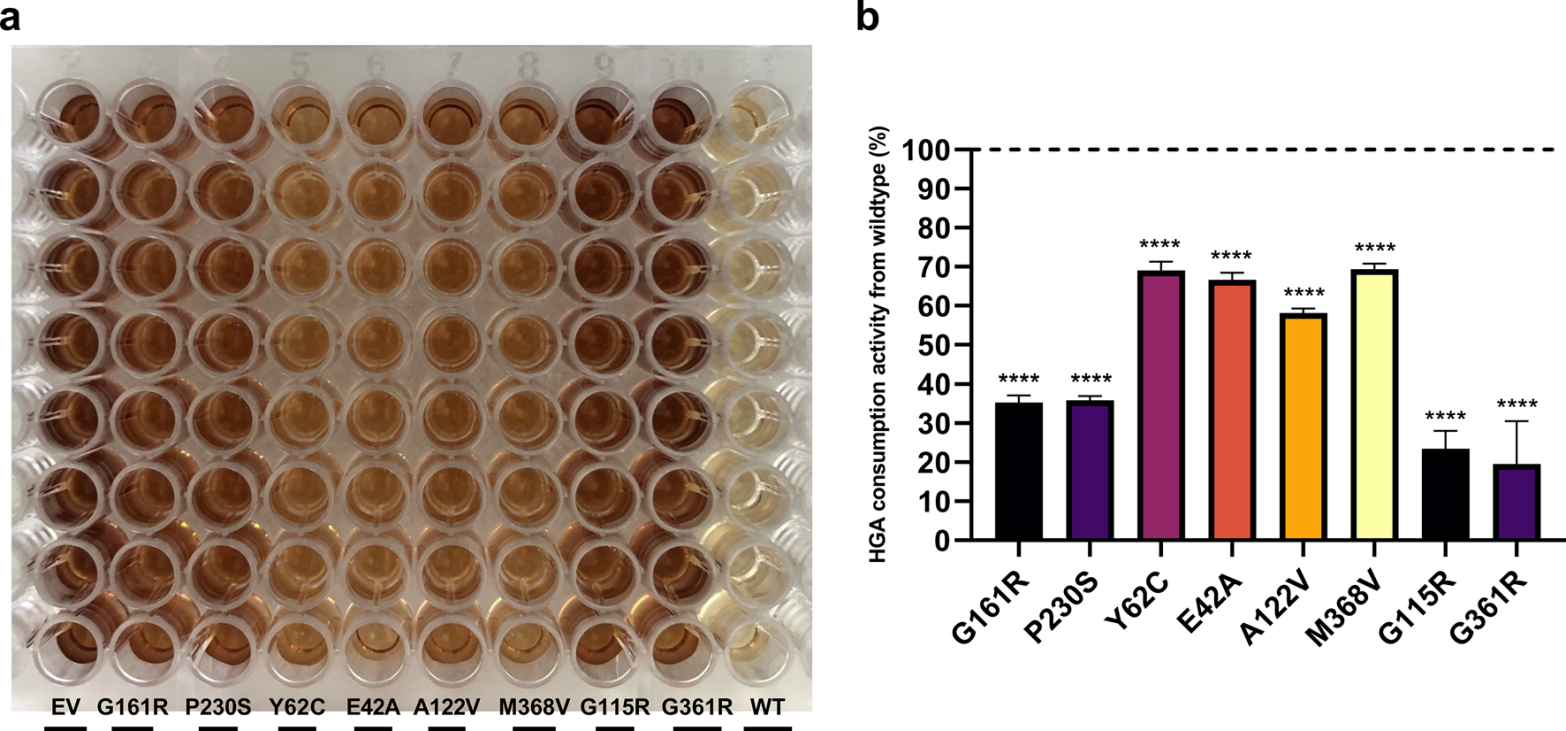
Evaluation of eight HGD missense variants based upon their residual HGD activities. Variants were generated via SDM and polymerase chain reaction (PCR) products were subsequently transformed into *E. coli* BL21 (DE3) cells. *E. coli* BL21 (DE3) cells containing pET42b-HGD WT, pET42b-HGD variants and the EV control were pre-grown and protein expression was induced as described in the Materials and Methods section. Simultaneously, 2.5 mM HGA was added to all cultures and a 96-well plate was filled according to (**a**) and incubated at expression temperature 30 °C. After 24 h incubation time, pyomelanin absorbance measurements were performed and residual HGD activities could be calculated following Eq. 1 (**b**). Significant differences compared to HGD WT control were calculated with a parametric one-way analysis of variance (ANOVA) followed by a Dunnett’s post-hoc test to correct for multiple comparisons. Data are expressed as mean ± standard deviation with ****p ≤ 0.0001. Calculated percentages of residual HGD activity were 70.37 ± 3.08% (for M368V), 68.78 ± 6.40% (for E42A), 58.15 ± 1.16% (for A122V), 69.07 ± 2.26% (for Y62C), 35.26 ± 1.90% (for G161R), 35.86 ± 1.14% (for P230S), 23.43 ± 4.63% (for G115R) and 19.57 ± 11.00% (for G361R). These results indicate that variant M368V resulted in the highest residual HGD activity followed by variants E42A, A122V, Y62C, G161R and P230S whereas variants G115R and G361R were found to be the least active variants. As a trend, one could observe that charged substitutions at positions 161, 115 and 361 seem to be less tolerable than substitutions to small hydrophobic amino acids at positions 368, 42 and 122.

To evaluate the functionality of our newly developed HTS assay, eight AKU causing missense variants (G161R, M368V, A122V, E42A, P230S, Y62C, G115R and G361R) were assessed on their ability to consume HGA. Therefore, an SDM was performed as described in the Materials and Methods section. *E. coli* BL21 (DE3) cells containing pET42b-HGD WT, pET42b-HGD variants and the EV were pre-grown and protein expression was induced as described in the Materials and Methods section. Thereafter, a 96-well plate was filled according to Fig. 7a. Subsequently, pyomelanin absorbance measurements of the supernatant fractions were performed and the percentage of residual HGD activity was calculated following Eq. 1. EV obtained OD_405_ of 2.24 ± 0.03 and was used as the Max signal, whereas HGD WT obtained OD_400_ of 0.532 ± 0.01 and was used as the Min signal. The calculated percentages of HGD activities from WT for each of the variants were 70.37 ± 3.08% (for M368V), 68.78 ± 6.40% (for E42A), 58.15 ± 1.16% (for A122V), 69.07 ± 2.26% (for Y62C), 35.26 ± 1.90% (for G161R), 35.86 ± 1.14% (for P230S), 23.43 ± 4.63% (for G115R) and 19.57 ± 11.00% (for G361R) (Fig. 7b). To conclude, the M368V variant resulted in the highest residual HGA consumption activity followed by variants E42A, A122V, Y62C, G161R and P230S, whereas variants G115R and G361R were found to be the least active variants.

## Discussion

AKU is an example of a rare monogenic autosomal-recessive disorder caused by a mutation in the HGD gene coding for the HGD hexameric protein, an enzyme involved in the tyrosine degradation pathway^7,34,47^. Several mutations within the HGD gene are known to be at the basis of AKU disease onset^14,37,40^. At present, 251 unique AKU-causing variants have been reported and summarized in the HGD mutation database^36^ from which 65% are missense mutations^11,17^. So far, the impact of these missense variants on residual HGD enzyme activities has not been quantified yet^37^. With the aim to bridge this knowledge gap, we present here an in vitro HTS system to rank AKU-causing missense variants based upon their ability to consume the oxidation-sensitive substrate HGA and the obtained results indicate that substitutions to positively charged Arg-residues have a stronger HGD activity reducing effect than substitutions to Ala or Val.

Previously, Rodriguez and colleagues^40^ evaluated the effects of a few AKU mutations on HGD enzyme function. Therefore, AKU-causing missense mutations were introduced by using an SDM protocol (QuikChange) and the appropriate mutagenic primers followed by sequencing to rule out undesired mutations. Then, His-tagged mutant HGD proteins were expressed in *E. coli* BL21 pLysS and lysed by the addition of lysis buffer. Subsequently, HGD variants were purified by Ni^2+^ affinity chromatography and kinetically characterized by their developed enzyme activity assay. However, lower residual HGD activities for the evaluated variants (A122V, M368V, E42A, P230S, Y62C and G161R) were found compared to those reported in our study^40^. This inconsistency can be due to the fact that during lysis and purification of the HGD enzymes, a fraction of the overexpressed HGD has become inactive. The latter was confirmed by another study where the purified fraction of WT HGD, expressed in *E. coli* BL21 (DE3) cells, accounts for only 40% of the initial activity of the lysed cells indicating an enzyme activity loss^48^. These findings in combination with the fact that missense variants have been shown to be associated with protein instabilities^49^, might be the reason for lower residual enzyme activities for some of the evaluated HGD variants. Moreover, when enzyme activity is measured according to Rodriguez et al. under aerobic conditions, the cofactor in its reduced state (Fe^2+^) has a higher affinity for HGD compared to its oxidized form. Therefore, in such preparations, active site iron is easily oxidized to Fe^3+^ resulting in lower enzyme activities^50^. However, our newly developed assay does not require this enzyme manipulation as upon the addition of IPTG and HGA to the cultures, the HGD enzyme immediately starts to convert HGA into MAA. Moreover, whole-cell biocatalysts allow for the internal supply of cofactors making the external addition of active site cofactor Fe^2+^ irrelevant^51^.

In our HTS assay, *E. coli* is used as preferred host organism for the overexpression of recombinant HGD due to its fast growth, low protein production costs and easy manipulation. However, solubility problems due to the formation of inclusion bodies, often occur at higher expression temperatures when *E. coli* is used as host organism^52,53^. The latter is due to the irreversible degradation that occurs if the protein is expressed at higher temperatures resulting in incorrect protein folding as a result of an increased translation rate^54^. When sub-optimal temperatures (between 15 and 30 °C) are used for heterologous protein expression, inclusion body formation is often reduced to a minimum. Moreover, lowering the cultivation temperature below 15 °C is not conventional as this might interfere with *E. coli* physiology and provoke a decrease in fatty acid saturation and inhibit DNA, RNA and protein synthesis^55,56^. Ideally, a compromise should be found between enzyme stability and optimal *E. coli* growth^54^. Therefore, in our optimized HTS assay, an expression temperature of 30 °C was used for HGD expression to prevent inclusion body formation. In addition, we observed that the auto-oxidation and self-polymerization process of HGA into pyomelanin was only complete when an expression temperature of 30 °C was used. Moreover, previous research on the kinetic characteristics of human HGD expressed in *E. coli* BL21 (DE3) cells, has demonstrated that no difference was seen in the ratio of inactive to active HGD when the protein was expressed at 30 °C^48^. Obviously, soluble, and active human HGD was observed in the supernatant fraction of *E. coli* BL21 (DE3) cells. This could be expected as human HGD is a globular protein characterized by hydrophilic residues at its surface that leads to the cytoplasmatic expression of HGD in human liver and kidney cells^25,57^. Our newly developed HTS assay provides a tool to study the effect of individual substitutions on K_cat_ and K_M_ as well as the effect of enzyme activity rescuing molecules to develop optimized treatments. A further important prerequisite would be to determine variant expression levels in patients since AKU patients can be homozygous or compound heterozygous^58,59^. In such instances, it is difficult to estimate the effect of each missense variant, as HGD’s quaternary structure can be formed either with monomers all affected by the same variant (homo-oligomer) or by two different ones (hetero-oligomer)^60^. Moreover, if mutations are located on two different alleles (trans), additive destructive effects might occur. Contrarily, if mutations belong to the same region (cis), effects could partially compensate^14^. However, our newly developed in vitro HTS system is not suitable to estimate the effect of compound heterozygosity in AKU patients as in bacteria it is not possible to express and evaluate ‘hybrid hexamers'. Therefore, the found residual enzyme activities in our HTS assay can differ from what is estimated in AKU patients. Refolding technologies have been described in literature to refold the protein of interest to its native, biologically active state and is initially an approach that has been suggested to resolubilize inclusion bodies derived from the overexpression of heterologous proteins in *E. coli* as host organism^61–63^. In general, first the protein is unfolded by applying an adequate level of force followed by the relaxation of this force resulting in protein refolding^64^. This alternative approach can be suggested to evaluate the effects of HGD hetero-oligomers obtained from combining two mutated purified enzymes followed by their denaturation and re-assembly. This might increase the chances to form hetero-oligomeric proteins with the aim to investigate in vitro the effects of compound heterozygosity for specific mutations in AKU patients. However, the renaturation step is often difficult to obtain and should ideally be optimized as the process depends on the conditions applied during the process; *i.e.* the refolding method, protein concentration, pH and temperature^63^. Recently, the REFOLDdb was created providing information on existing protein refolding protocols, but, unfortunately, human HGD has not yet been included in this database indicating that this process should be optimized first^63^. Over the past years, HTS systems have become particularly interesting in the field of drug discovery. First of all, they provide a resource to identify new potential therapeutics while on the other hand they tend to gain insight into biological processes. Such systems demand an extensive validation prior to implementation^65^. Therefore, this HTS assay has been developed with the aim to evaluate missense variants in the context of AKU and was evaluated on robustness and reliability aiming for assay validation according to the NCGC guidelines^44^. Average plate Z’ factor, a widely-used assay quality parameter^66^, was 0.88 ± 0.02 in 96-well plate format and passed the minimum acceptance criteria for Z’ factor ≥ 0.40 when only two repeats per variant are used. A possible limitation of our developed HTS assay is that the system is designed in bacteria and not in human cell lines and can therefore not be considered as the ‘real life’ situation indicating that the found residual enzyme activities in our HTS assay can differ from what is estimated in AKU patients. Moreover, in AKU patients, far lower circulating HGA concentrations are found^10,24^. Although, if lower HGA concentrations would be used in our developed HTS assay, the assay quality parameters (Z’ and SW) would tremendously decrease. In that case, the separation of the assay control data (difference between HGD WT and EV signals) would significantly reduce making it impossible to reliably distinguish the tested HGD variants and this will finally result in a poor assay quality. However, we have proven that our HTS assay can be used as a reliable tool to compare the enzyme activities of human HGD variants. Moreover, we have developed a system that is fast, cost-effective, reliable, and simple. In addition, it provides a tool to fill the knowledge gap on the estimation of residual enzyme activities of AKU-causing missense variants based on their intrinsic activity to prevent the formation of pyomelanin which is of high clinical relevance. On a final note, this assay may contribute to the identification and development of novel treatment strategies for selected variants in AKU.

## Methods

### Materials

All chemicals were of analytical grade or higher and purchased from Sigma-Aldrich Chemie GmbH (Schnelldorf, Germany). OD_600_ measurements were performed by an Eppendorf Biophotometer D30 (Hamburg, Germany). Protein expression was performed in MTP (96-wells, conical bottom) purchased from Greiner Bio-One GmbH (Kremsmünster, Austria) and thereafter incubated in an Eppendorf New Brunswick™ Innova 42 R incubator shaker series (Hamburg, Germany). Pyomelanin absorbance measurements were performed by a VICTOR 3 1420 Multilabel Counter plate reader (Perkin Elmer, Waltham, MA, USA). Plasmid isolation kits were obtained from Sigma-Aldrich Chemie GmbH (Schnelldorf, Germany). PCRs for SDM were performed with Icycler IQ5 (Bio-Rad). Mastermix reagents were purchased from Thermo Fisher Scientific (Waltham, MA, USA).

### Bacterial strains, plasmids and target gene

cDNAs of WT human HGD (NM_000187.3) were purchased from GeneArt in standard pMA-based cloning plasmids (Thermo Scientific), codon-optimized for *E. coli* (Supplementary information 2) and subsequently subcloned using restriction enzymes (NdeI-EcoRI) by ligase-based cloning in the multiple cloning site (MCS) of pET42b(+) vectors. To ensure high-level protein expression and purification in *E. coli* bacteria, pET42b(+) vectors were used from Novagen (Darmstadt, Germany) and selection pressure was maintained by the presence of a kanamycin (KANA) resistance gene. Lysogeny Broth (LB) medium supplemented with or without 1% glucose, was used as the standard growth medium supplemented with KANA (50 μg/mL). *E. coli* DH5α strain (Stratagene, La Jolla, CA, USA) was used as host for maintenance and propagation. For expression of the human HGD transgene, *E. coli* BL21 (DE3) (Agilent Technologies, Santa Clara, CA, USA) and *E. coli* C43 (DE3) (Lucigen Corporation, Middleton, WI, USA) strains were used. As high signal for pyomelanin accumulation, a pET42b(+) plasmid without transgene was used and was obtained by heat shock transformation into *E. coli* which is hereafter called the EV.

### Selection of optimal *E. coli* strain and expression temperature for WT HGD

In order to select an ideal *E. coli* strain for reliable expression of human HGD, both *E. coli* strains BL21 (DE3) and C43 (DE3) were compared. After heat shock transformation with pET42b-HGD, *E. coli* cultures of both strains were inoculated in LBG_KANA_ and incubated overnight at 37 °C and 250 rpm in a shaking incubator. Overnight *E. coli* cultures were diluted in fresh LB_KANA_ medium until OD_600_ of 0.1 and subsequently incubated at 30 °C and 250 rpm until an OD_600_ of 0.6–0.8 was reached. HGD protein expression was induced by the addition of 1 mM IPTG and cultures were further incubated at different expression temperatures (22 °C, 30 °C and 37 °C; 250 rpm). Samples for protein extraction were taken at regular time points until 48 h post-IPTG induction. Experiments were performed independently from each other in triplicate.

### Protein extraction, SDS-PAGE and western blot analysis

Protein extraction was performed by isolating samples at regular time intervals as described previously. First of all, OD_600_ measurements were performed for each sample to normalize for bacterial cell concentration and samples were thereafter centrifuged (5 min, 3200 g, 4 °C). Bacterial pellets were washed in cool and sterile HEPES buffer (20 mM, pH 7.0) and subsequently centrifuged (2 min, 2348 g, RT). Supernatant fractions were discarded, and pellets were frozen until further use. Next, samples were prepared for western blot analysis. Therefore, pellets were resuspended in 485 µL HEPES buffer (20 mM, pH 7.0) and sonicated (10 s pulse time, 1 min., amplitude 40%) for complete fragmentation of cellular membranes. Lysed samples were centrifuged (10 min, maximum speed, 4 °C) to separate supernatant from pellet fractions and soluble proteins in supernatant fractions were analyzed via western blot. Therefore, protein samples were first separated via sodium dodecyl sulfate (SDS) polyacrylamide gel electrophoresis (PAGE) on a 12% *(w/v)* Mini-Protean® TGX Stain-free™ gel (Bio-rad, Temse, Belgium) and thereafter immediately blotted onto a nitrocellulose Trans-Blot® Turbo™ transfer packs (Bio-rad, Temse, Belgium). Next, membranes were blocked for one hour in blocking buffer containing 5% *(m/v)* milk powder in Tris-buffered saline solution (*i.e.*, 20 mM Tris–135 mM NaCl) containing 0.1% *(v/v)* Tween-20 and overnight incubated at 4 °C with primary polyclonal anti-HGD antibody (HPA052359) produced in rabbit (Sigma-Aldrich, Saint-Louis, MO, USA) diluted in 1:1000 ratio in blocking buffer. Membranes were once more incubated at room temperature for one hour with polyclonal secondary goat anti-rabbit antibody (Dako, Agilent Technologie, Heverlee, Belgium) diluted in 1:1000 ratio in blocking buffer and washed to remove redundant antibody with Tris-buffered saline solution containing 0.1% *(v/v)* Tween-20 in between incubation steps. Signals were detected by using the Pierce™ enhanced chemiluminescence substrate (ECL) Western Blotting substrate kit (Thermo Scientific, Rockford, IL, USA) and ChemiDoc MP imaging system (Bio-rad, Belgium). HGD protein bands were quantified by means of Bio-Rad ImageLab software (v. 5.2.1) and pixel density data was normalized by subtracting the average signal of three background areas from our sample data.

### Identify time point for complete auto-oxidation and self-polymerization of HGA into pyomelanin

LBG_KANA_ was inoculated with *E. coli* BL21 (DE3) cells containing pET42b-HGD plasmid and the EV control and thereafter transferred into a shaking incubator for overnight incubation at 37 °C and 250 rpm. Overnight *E. coli* cultures were diluted in fresh LB_KANA_ medium until OD_600_ of 0.1 and further incubated at 30 °C and 250 rpm until OD_600_ of 0.6 was reached. Thereafter, both cultures were supplemented with 2.5 mM of HGA substrate and further incubated at 22 °C and 30 °C during 50 h. Pyomelanin absorbance measurements were performed at regular time intervals until 50 h post-IPTG addition.

### Dose response experiment to select ideal HGA concentration

LBG_KANA_ was inoculated with *E. coli* BL21 (DE3) cells containing pET42b-HGD plasmid and the EV control and thereafter transferred into a shaking incubator for overnight incubation at 37 °C and 250 rpm. Overnight *E. coli* cultures were diluted in fresh LB_KANA_ medium until OD_600_ of 0.1 and further incubated at 30 °C and 250 rpm until OD_600_ of 0.6 was reached. Both cultures were subsequently supplemented with different HGA concentrations ranging from 0 to 5 mM with four replicates per concentration and further incubated at 30 °C in a deep-well 96 well-plate. Pyomelanin absorbance was measured after 24 h and 36 h post-IPTG addition.

### Optimal expression conditions

For optimal *E. coli* growth, LBG_KANA_ was inoculated and thereafter transferred into a shaking incubator for overnight incubation at 37 °C and 250 rpm. Overnight *E. coli* cultures were diluted in fresh LB_KANA_ medium until OD_600_ of 0.1 and further incubated at 30 °C and 250 rpm until OD_600_ of 0.6 was reached. HGD protein expression was induced by the addition of 1 mM IPTG. Subsequently, *E. coli* cultures were supplemented with 2.5 mM HGA and cells were further incubated at 30 °C and 250 rpm for another 24 h to induce complete pyomelanin formation.

### Generation of HGD missense variants via SDM

cDNA of WT HGD-pET42b was isolated from recombinant *E. coli* DH5α by using the plasmid DNA miniprep kit according to manufacturer’s manual (Sigma-Aldrich Chemie GmbH, Schnelldorf, Germany). Primers were designed (Supplementary information 3) to substitute a single amino acid within the HGD sequence to create the following variants: G161R, M368V, A122V, E42A, P230S, Y62C, G115R and G361R responsible for AKU’s outcome. Pre-PCR master mixes (MM) (50 µL) for a single-primer extension stage contained 20 ng cDNA, 10 µL 5 × High-Fidelity Phusion buffer, 400 nM forward primer or reversed primer, 0.2 mM dNTP-mix, Phusion High-Fidelity DNA polymerase (0.02 U/µL) and UltraPure DNAse RNAse free destilled water (all from ThermoScientific) to reach a final volume of 50 µL for each sample. The following temperature cycling conditions were used: denaturation for 30 s at 98 °C followed by 3 cycles of 98 °C for 10 s, annealing at X for 30 s and elongation at 72 °C for 60 s. Subsequently, equal amounts of forward and reversed reaction tubes were combined and again ran with the following temperature cycling conditions: 30 s at 98 °C followed by 15 cycles of 98 °C for 10 s, X for 30 s, 72 °C for 60 s and 72 °C for 10 min for final elongation. Annealing temperatures for both PCR programs were 55 °C for variants G161R and M368V; 52.4 °C for variant A122V; 66 °C for variants E42A, P230S, Y62C and 65 °C for variants G115R and G361R. Subsequently, PCR products were analyzed for successful amplification. Agarose gels were prepared in a final concentration of 1% *(w/v)* in 1 × Tris–acetate-EDTA (TAE) buffer and stained with a fluorescent Nucleic Acid Stain GelRed® (Biotum, Russia) in 1:10,000 dilution. Samples were prepared by the addition of 6 × DNA Loading Dye (Thermo Scientific, Waltham, MA, USA) to each sample. PCR products were loaded into the wells of the gel and the electrophoresis tank was filled with 1 × TAE buffer. GeneRuler 1 kb DNA ladder (Thermo Fisher Scientific, Waltham, MA, USA) was loaded into the first well. The gel ran at 100 V and 300 mA for one hour and was visualized by ChemiDoc MP imaging system (Bio-rad, Temse, Belgium). After confirmation of successful amplification, PCR products were DpnI digested at 37 °C during 30 min (ThermoScientific) (1 µL/50 µL MM) for restriction of methylated template DNA and the restriction enzyme was subsequently heat inactivated at 80 °C for 20 min. PCR products were thereafter heat shock transformed into *E. coli* BL21 (DE3) and HGD variants were Sanger sequenced (Eurofins Genomics, Germany) after plasmid preparation to confirm their respective point mutation.

### Variant analysis

For the HTS assay, *E. coli* cultures containing pET42b-HGD variants were induced when an OD_600_ of 0.6–0.8 was reached by supplementation with 1 mM IPTG in LB_KANA_ medium. Each culture was supplemented with 2.5 mM HGA and subsequently 600 μL of culture was added to each well of a 96-well conical bottom MTP with 8 replicates per HGD variant and columns 1 and 12 of the 96-well plate were left empty due to the significant edge effect. After incubation (400 rpm, 24 h, 30 °C), plates were centrifuged (5810R, Eppendorf, Hamburg, Germany) at 3200 g for 10 min. For pyomelanin absorbance measurements, 150 μL supernatant of each well was transferred into a 96-well flat bottom MTP (Thermo Scientific) and absorbance was measured at a wavelength of 405 nm by a plate reader. Residual HGD activities (% Res. Act.) were subsequently calculated according to the following equation:

1 $$ \%\,\text{Res. Act.}= { }\left[ {1 - { }\left( {\frac{{{\text{OD}}_{405} {\text{ VAR }} - {\text{ OD}}_{405} {\text{WT}}}}{{{\text{OD}}_{405} {\text{ EV}} - {\text{ OD}}_{405} {\text{ WT}}}} } \right)} \right]{\text{ x }}100$$

where OD405 VAR = measured OD405 of the measured HGD missense variant, OD405 WT = measured OD405 of HGD WT (low signal), OD405 EV = measured OD405 of the EV (high signal). Residual HGD activity values were calculated with Graphpad Prism (v. 9.4.0). Significant differences compared to HGD WT control were calculated with a parametric one-way analysis of variance (ANOVA) followed by a Dunnett’s post-hoc test to correct for multiple comparisons.

### Robustness of the assay

A plate uniformity study was performed on three different days at minimum (HGD WT), medium (M368V) and maximum (EV) signal in triplicate (3 days, 3 plates). The high and low signal were derived from the EV and HGD WT culture, respectively, whereas the middle signal was derived from M368V with approximately 37% residual HGD activity according to literature. On three different days, the same plate lay-out was used. According to a certain plate lay-out (plate 1: HML, plate 2: LHM, plate 3: MLH), a volume of 150 μL of each well was transferred into a 96-well flat bottom MTP. Absorbance measurements and protein expression parameters were performed as explained above. Based upon the pyomelanin absorbance measurements; mean, SD, CV, SW and Z’ factors were calculated. The Z’ factor and SW were calculated according to the following equations:

2 $${\text{Z}}\prime = \frac{{\left( {{\text{AVG}}_{{{\text{Max}}}} - 3 \frac{{{\text{SD}}_{{{\text{Max}}}} }}{{\sqrt {\text{n}} }}} \right) - \left( {{\text{AVG}}_{{{\text{Min}}}} - 3 \frac{{{\text{SD}}_{{{\text{Min}}}} }}{{\sqrt {\text{n}} }}} \right)}}{{{\text{AVG}}_{{{\text{Max}}}} - {\text{AVG}}_{{{\text{Min}}}} }}$$

3 $${\text{SW }} = \frac{{\left( {{\text{AVG}}_{{{\text{Max}}}} - 3{ }\frac{{{\text{SD}}_{{{\text{Max}}}} }}{{\sqrt {\text{n}} }}} \right) - \left( {{\text{AVG}}_{{{\text{Min}}}} + { }3{ }\frac{{{\text{SD}}_{{{\text{Min}}}} }}{{\sqrt {\text{n}} }}} \right)}}{{\frac{{{\text{SD}}_{{{\text{Max}}}} }}{{\sqrt {\text{n}} }}}}$$

where n is the number of HGD variant replicates used for the HTS assay.

## Supplementary Information

Supplementary Information.

## Data Availability

The data generated or analyzed during this study are included in this published article (and its supplementary information files). The genetic polymorphisms sequence data that supports the findings of this study have been deposited in repository dbSNP ( https://www.ncbi.nlm.nih.gov/snp/) with the following accession codes: rs120074173 (for M368V), rs373921680 (for E42A), (for A122V), rs1174584850 (for Y62C), rs28941783 (for G161R), rs28942100 (for P230S), rs755734596 (for G115R) and rs765219004 (for G361R).
