# Supplementary material for: A robust bacterial high-throughput screening system to evaluate single nucleotide polymorphisms of human homogentisate 1,2-dioxygenase in the context of alkaptonuria
Source: Sci Rep. 2022 Nov 14;12:19452. doi: 10.1038/s41598-022-23702-y (PMC9663557; doi:10.1038/s41598-022-23702-y)
Supplement: Supplementary file 1 — Supplementary Information. [file 41598_2022_23702_MOESM1_ESM.pdf]

# A robust bacterial high-throughput screening system to evaluate single nucleotide polymorphisms of human homogentisate 1,2-dioxygenase in the context of alkaptonuria

**Sien Lequeue[1,*], Jessie Neuckermans[1], Ine Nulmans[1], Ulrich Schwaneberg[2], Tamara Vanhaecke[3], Joery De Kock[1,*]**

[1] Vrije Universiteit Brussel, Liver Therapy & Evolution team, *In Vitro* Toxicology and Dermato-Cosmetology (IVTD) research group, Faculty of Medicine and Pharmacy, Laarbeeklaan 103, B-1090 Brussels, Belgium.

[2] RWTH Aachen University, Lehrstuhl für Biotechnologie, Worringerweg 3, 52074 Aachen, Germany.

[3] Vrije Universiteit Brussel, *In Vitro* Liver Disease Modelling team, *In Vitro* Toxicology and Dermato-Cosmetology (IVTD) research group, Faculty of Medicine and Pharmacy, Laarbeeklaan 103, B-1090 Brussels, Belgium.

## Supplementary information

**Supplementary Fig. S1.** Protein expression of human HGD was evaluated in both *E. coli* BL21 (DE3) **(a)** and *E. coli* C43 (DE3) **(b)** strains and for different expression temperatures (22°C, 30°C and 37°C) by western blot analysis between 0 and 48 h post-IPTG addition. Here, the original uncropped blots are shown. For both *E. coli* strains, a molecular weight band at 49 kDa was observed in the supernatant fractions for the three assessed temperatures from one hour after IPTG addition onwards. Human HGD expression was observed to be higher in *E. coli* BL21 (DE3) compared to *E. coli* C43 (DE3). Expression temperatures 22°C and 30°C achieved the highest and most stable protein expression in both strains, while HGD was only mediocrely expressed at 37°C.

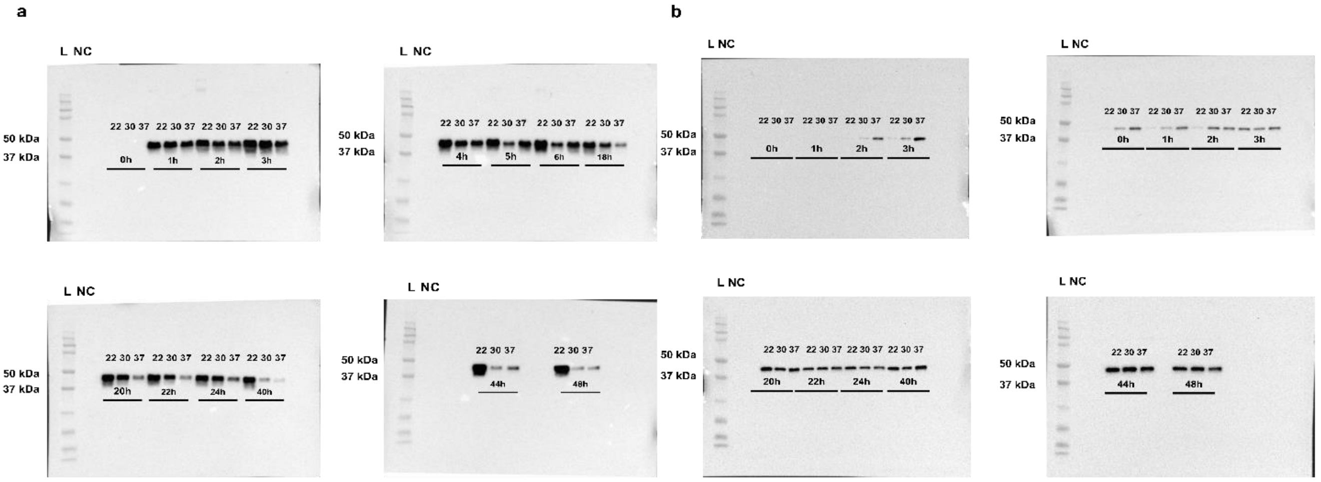

**Supplementary information 2: HGD cDNA codon-optimized for *E. coli*.**

ATGGCCGAGCTGAAATATATCAGCGGTTTTGGTAATGAATGCAGCAGCGAAGATCCGCGTTGTCCG
GGTAGCCTGCCGGAAGGTCAGAATAATCCGCAGGTTTGTCCGTATAATCTGTATGCAGAACAGCTG
AGCGGTAGCGCATTTACCTGTCCGCGTAGCACCAATAAACGTAGCTGGCTGTATCGTATTCTGCCG
AGCGTTAGCCATAAACCGTTTGAAAGCATTGATGAAGGTCAGGTTACCCATAATTGGGATGAAGTT
GATCCTGATCCGAATCAGCTGCGTTGGAAACCTTTTGAAATTCCGAAAGCAAGCCAGAAAAAAGTG
GATTTTGTTAGCGGTCTGCATACCCTGTGTGGTGCCGGTGATATCAAAAGCAATAATGGTCTGGCC
ATCCATATCTTTCTGTGTAATACCAGCATGGAAAATCGCTGCTTTTATAACAGTGATGGCGATTTTC
TGATTGTGCCGCAGAAAGGTAATCTGCTGATTTATACCGAATTTGGCAAAATGCTGGTTCAGCCGA
ATGAAATTTGTGTTATTCAGCGTGGTATGCGCTTTAGCATCGATGTTTTTGAAGAAACCCGTGGCTA
TATCCTGGAAGTTTATGGTGTTCATTTTGAACTGCCGGATCTGGGTCCGATTGGTGCAAATGGCCTG
GCAAATCCGCGTGATTTCCTGATTCCGATTGCATGGTATGAAGATCGTCAGGTTCCGGGTGGTTATA
CCGTTATTAACAAATATCAGGGCAAACTGTTTGCAGCCAAACAGGATGTTAGCCCGTTTAATGTTG
TTGCATGGCATGGTAATTATACCCCGTATAAATACAACCTGAAAAACTTCATGGTGATCAACAGCG
TTGCATTTGATCATGCAGATCCGAGCATTTTTACCGTTCTGACCGCAAAAAGCGTGCGTCCGGGTGT
TGCAATTGCCGATTTTGTGATTTTTCCGCCTCGTTGGGGTGTTGCCGATAAAACCTTTCGTCCGCCTT
ATTATCATCGTAATTGCATGAGCGAGTTTATGGGTCTGATTCGTGGTCATTATGAAGCAAAACAGG
GTGGTTTTCTGCCTGGTGGTGGTAGTCTGCATAGCACCATGACACCGCATGGTCCGGATGCAGATT
GTTTTGAAAAAGCCAGCAAAGTTAAACTGGCACCGGAACGTATTGCAGATGGCACCATGGCATTTA
TGTTTGAAAGTAGCCTGAGCCTGGCAGTTACCAAATGGGGTCTGAAAGCCAGCCGTTGTCTGGATG
AAAATTATCATAAATGTTGGGAGCCGCTGAAAAGCCATTTTACCCCGAATAGCCGTAATCCGGCAG
AACCGAATTAA

**Supplementary Information 3: Site-directed mutagenesis (SDM) primers.**

**SDM HGD A122V**

*FW primer A122V*:

5'- CCT GTG TGG TGT CGG TGA TAT C -3'

*RV primer A122V:*

5'- GAT ATC ACC GAC ACC ACA CAG G -3'

**SDM HGD G161R**

*FW primer G161R:*

5'- GAT TGT GCC GCA GAA ACG TAA TCT GCT G -3'

*RV primer G161R:*

5'- CAG CAG ATT ACG TTT CTG CGG CAC AAT C -3'

**SDM HGD M368V**

*FW primer M368V:*

5'- CAT AGC ACC GTG ACA CCG CAT G -3'

*RV primer M368V:*

5'- CAT GCG GTG TCA CGG TGC TAT G -3'

**SDM HGD Y62C**

*FW primer Y62C:*

5'- GTA GCT GGC TGT GTC GTA TTC TGC -3'

*RV primer Y62C:*

5'- GCA GAA TAC GAC ACA GCC AGC TAC -3'

**SDM HGD E42A**

*FW primer E42A:*

5'- CTG TAT GCA GCA CAG CTG AGC -3'

*RV primer E42A:*

5'- GCT CAG CTG TGC TGC ATA CAG -3'

**SDM HGD P230S**

*FW primer P230S:*

5'- GAT TTC CTG ATT TCG ATT GCA TGG TAT G -3'

*RV primer P230S:*

5'- CAT ACC ATG CAA TCG AAA TCA GGA AAT C -3'

**SDM HGD G115R**

*FW primer G115R:*

5'- TTT GTT AGC CGT CTG CAT ACC -3'

*RV primer G115R:*

5'- GGT ATG CAG ACG GCT AAC AAA -3'

**SDM HGD G361R**

*FW primer G361R:*

5'- CTG CCT GGT CGT GGT AGT CTG-3'

*RV primer G361R:*

5'- CAG ACT ACC ACG ACC AGG CAG -3'
